# Evaluating the Use of Generative Artificial Intelligence to Support Genetic Counseling for Rare Diseases

**DOI:** 10.3390/diagnostics15060672

**Published:** 2025-03-10

**Authors:** Suok Jeon, Su-A Lee, Hae-Sun Chung, Ji Young Yun, Eun Ae Park, Min-Kyung So, Jungwon Huh

**Affiliations:** 1 Department of Genetic Counseling, Graduate School, College of Medicine, Ewha Womans University, Seoul 03760, Republic of Korea; suok12345@gmail.com (S.J.); sualee91@gmail.com (S.-A.L.); 2 Department of Laboratory Medicine, College of Medicine, Ewha Womans University, Seoul 03760, Republic of Korea; sunny0521.chung@ewha.ac.kr; 3 Department of Neurology, College of Medicine, Ewha Womans University, Seoul 03760, Republic of Korea; movement@ewha.ac.kr; 4 Department of Pediatrics, College of Medicine, Ewha Womans University, Seoul 03760, Republic of Korea; pea8639@ewha.ac.kr

**Keywords:** generative artificial intelligence, genetic counseling, rare diseases

## Abstract

**Background/Objectives**: Rare diseases often present challenges in obtaining reliable and accurate information than common diseases owing to their low prevalence. Patients and families often rely on self-directed learning, but understanding complex medical information can be difficult, increasing the risk of misinformation. This study aimed to evaluate whether generative artificial intelligence (AI) provides accurate and non-harmful answers to rare disease-related questions and assesses its utility in supporting patients and families requiring genetic counseling. **Methods**: We evaluated four generative AI models available between 22 September and 4 October 2024: ChatGPT o1-Preview, Gemini advanced, Claude 3.5 sonnet, and Perplexity sonar huge. A total of 102 questions targeting four rare diseases, covering general information, diagnosis, treatment, prognosis, and counseling, were prepared. Four evaluators scored the responses for professionalism and accuracy using the Likert scale (1: poor, 5: excellent). **Results**: The average scores ranked the AI models as: ChatGPT (4.24 ± 0.73), Gemini (4.15 ± 0.74), Claude (4.13 ± 0.82), and Perplexity (3.35 ± 0.80; *p* < 0.001). Perplexity had the highest proportion of scores of 1 (very poor) and 2 (poor) (7.6%, 31/408), followed by Gemini (2.0%, 8/408), Claude (1.5%, 6/408), and ChatGPT (1.5%, 6/408). The accuracy of responses in the counseling part across all four diseases was significantly different (*p* < 0.001). **Conclusions**: The four generative AI models generally provided reliable information. However, occasional inaccuracies and ambiguous references may lead to confusion and anxiety among patients and their families. To ensure its effective use, recognizing the limitations of generative AI and providing guidance from experts regarding its proper utilization is essential.

## 1. Introduction

Rare diseases pose significant challenges owing to the difficulty in early diagnosis and lack of effective treatments or therapeutic drugs, exacerbating the economic and psychological burden on patients and their families. Their rarity often limits the availability of comprehensive disease information, leading to national and international efforts to improve policies and support systems. For example, in South Korea, the Korea Disease Control and Prevention Agency (KDCA) has established a Rare Disease Helpline (https://helpline.kdca.go.kr/cdchelp/ accessed on 1 December 2024) in 2006. This platform provides information on rare diseases, orphan drugs, specialized medical institutions, financial support programs, and genetic testing. Despite these initiatives, interactive professional counseling among patients and their families is in demand [1]. However, the current healthcare system in South Korea struggles to provide genetic counseling as a part of its medical services. The prevalence of so-called “five-minute consultations” renders conducting thorough family history reviews or counseling for genetic diseases nearly impossible. This limitation highlights the need for professionally trained genetic counselors. A survey of 102 experts, including members of the Korean Society of Medical Genetics and Genomics, genetic testing organizations, and hospitals treating patients with genetic diseases, revealed that 88% of experts acknowledged the necessity for systematic genetic counseling [2]. Conversely, another survey of 185 patients with rare diseases and their caregivers found that 79.8% of participants did not receive genetic counseling [3].

In response to these gaps, patients and their families often rely on self-directed learning through books or Internet searches, and information exchange via online and offline patient communities. However, understanding and validating this information without a genetic background can be challenging and carries the risk of misinformation. This issue is particularly pronounced for ultra-rare diseases for which the global number of patients is extremely low and information is limited.

Amidst these challenges, the rapid development and widespread adoption of generative artificial intelligence (AI) globally offers promising potential for improving access to disease-related information. AI, which is defined as a technology that enables computers or machines to perform tasks that typically require human intelligence, has evolved significantly with the emergence of generative AI. This advanced form of AI actively generates outcomes such as data or content in response to specific user queries by autonomously searching for, and learning from, relevant data [4]. According to the Korea Information Society Development Institute, 12% of South Koreans were already utilizing generative AI in 2023 for information retrieval, conversation, work assistance, and learning support [5].

Research on AI has grown exponentially since the 2010s, with publications doubling over 12 years by 2021 [6]. AI is recognized as a general-purpose technology integrated into industries such as manufacturing, healthcare, education, and finance [7]. Healthcare and medical fields have received the highest AI investment in 2022, reflecting its transformative role in this sector [6]. Concurrently, concerns regarding the fairness, transparency, and information accuracy of AI have emerged as critical research areas. However, generative AI systems have limitations in healthcare applications, occasionally producing fabricated data or incorrect diagnoses, which can lead to potential misinformation [6]. Another study has highlighted the inherent dependence of AI on digital data, which can perpetuate biases, fail to detect errors, or trigger cascading effects with adverse outcomes [8]. These findings underscore the risk of inaccurate information and potential harm if users fail to identify such errors.

Genetic counseling involves sensitive information, including genetic data, family history, and cultural, ethical, or religious considerations, and it extends beyond the individual to encompass families and broader communities. Therefore, delivering neutral and accurate information is crucial.

This study evaluated four leading generative AI models using the Korean language, selected based on global usage rankings at the time of the research, to assess their ability to provide accurate information in response to rare-disease-related queries. Furthermore, we explored whether these generative AI systems could support patients and families in need of genetic counseling.

## 2. Materials and Methods

### 2.1. Generative AI Model Selection

Four generative AI models, ChatGPT o1-Preview (ChatGPT; OpenAI, San Francisco, CA, USA), Gemini Advanced (Gemini; Google DeepMind, Mountain View, CA, USA), Claude 3.5 sonnet (Claude; Anthropic, San Francisco, CA, USA), and Perplexity sonar (Huge model) (Perplexity; Perplexity AI, San Francisco, CA, USA), were selected for evaluation based on their high global user rankings and suitability for disease-related query–response tasks (Table S1). This study utilized the highest-tier versions of these models available during the research period (22 September–4 October 2024). For Perplexity, its “Web mode” was specifically utilized among its multiple exploration modes.

### 2.2. Rare Disease Selection

Four rare diseases were selected based on the number of newly registered cases in South Korea within the rare and ultra-rare disease categories, as well as chromosomal disorders from 2020 to 2021. Data were obtained from the Rare Disease Registry Statistics published by the KDCA [9]. The selected diseases demonstrated diverse characteristics, including variations in onset age, genetic etiology, diagnostic methods, treatments, and prognoses, to evaluate the ability of generative AI models to provide appropriate responses to varied queries (Table S2).

### 2.3. Development of Evaluation Questions and Metrics for Generative AI Responses

Based on the selected rare diseases, standardized questions were designed to evaluate the accuracy of information provided by the generative AI models in the Korean language. To ensure consistency across diseases and identify trends in AI responses, five subcategories—general information, diagnosis, treatment, prognosis, and counseling—were established, with 22–29 questions crafted for each disease.

Accordingly, three evaluation criteria were established to assess generative AI responses: (1) professionalism, (2) information accuracy, and (3) impact on patients and their families.

A Likert scale ranging from 1 (very poor) to 5 (excellent) was used to evaluate each response comprehensively (Table S3).

### 2.4. Study Procedure

A total of 102 standardized questions were sequentially presented to each generative AI model, and their responses were compiled in Excel for analysis, resulting in 408 evaluations per evaluator. The evaluation team comprised two professors and two postgraduate students specializing in genetic counseling. Evaluations were conducted over 5 weeks (29 September–2 November 2024), with anonymized AI responses to ensure impartiality. The evaluators worked independently until all the scores were submitted.

To ensure fairness, items with a score discrepancy of two points or more among evaluators were reviewed, with evaluators providing justifications and revising their scores, if necessary. Items with an average score below 3.00 were further reviewed, along with the evaluators’ comments. Items rated as “poor” (2 or below) by some evaluators but “satisfactory” (3 or above) by others were reassessed regardless of the score gap. In total, 38 items were re-evaluated, leading to final adjustments for 30 items. This study was exempt from review by the Institutional Review Board of Ewha Womans University (IRB No. ewha-202409-0006-01).

### 2.5. Statistical Analysis

Data were analyzed using SPSS Statistics 27.0 (SPSS; IBM, Armonk, NY, USA). Differences in the mean scores among evaluators were assessed using the Friedman test. The Kruskal–Wallis H test was employed to analyze the differences in response accuracy across specific diseases and AI models, with *p* < 0.05 considered statistically significant. Post hoc analyses using Bonferroni-adjusted *p*-values were conducted to confirm the significant findings.

## 3. Results

### 3.1. Comparison of Total Scores Across Generative AI Models

When comparing the evaluation scores of the generative AI models, the average scores were ranked in the following order: ChatGPT, Gemini, Claude, and Perplexity, with significant differences among the models (*p* < 0.001; Table 1). Post-hoc analysis with Bonferroni-adjusted *p*-values confirmed that perplexity’s average score was significantly lower than that of the other models (*p* < 0.001; Table 2).

### 3.2. Comparison of Subcategory Scores by Disease for Generative AI Models

**Figure 1:**
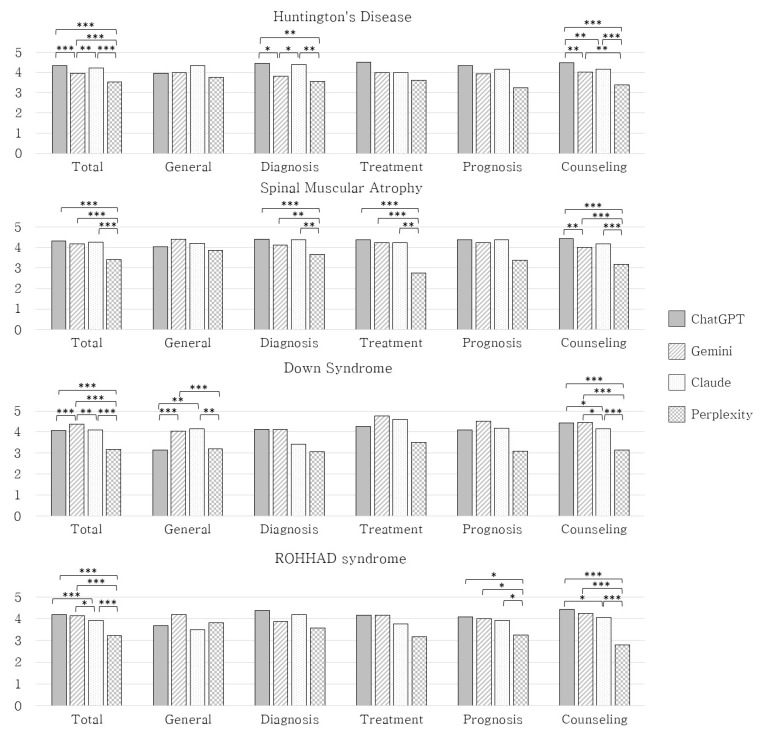
Comparison of generative AI model score across different subcategories in different diseases. * *p* < 0.05, ** *p* < 0.01, *** *p* < 0.001.

A total of 408 evaluations across 102 questions related to four rare diseases were analyzed for score distributions among the generative AI models. Perplexity, Gemini, Claude, and ChatGPT received scores of 1 (very poor) or 2 (poor) in 7.6% (31/408), 2.0% (8/408), 1.5% (6/408), and 1.5% (6/408) cases, respectively. Conversely, ChatGPT, Gemini, Claude, and Perplexity achieved scores of 4 (good) or 5 (excellent) in 85.5% (349/408), 82.8% (338/408), 78.2% (319/408), and 36.0% (147/408) cases, respectively. The Likert-scale distributions for each AI model across all questions are presented in Table S4. When comparing mean scores by subcategory for each disease, significant differences were observed in the “Counseling” subcategory across all four diseases (*p* < 0.001). Additionally, for Huntington’s disease, significant differences were noted in the “Diagnosis” subcategory (*p* = 0.018). For spinal muscular atrophy, significant differences were observed in both the “Diagnosis” (*p* = 0.004) and “Treatment” (*p* = 0.037) subcategories. For Down syndrome, significant differences were found in the “General” subcategory (*p* = 0.006). For ROHHAD syndrome, significant differences were identified in the “Prognosis” subcategory (*p* = 0.048). Post hoc analyses using the Wilcoxon signed-rank test revealed these differences (Figure 1). Detailed score comparisons for each subcategory are presented in Table S4.

### 3.3. Analysis of Responses with an Average Score Below 3 Across Generative AI Models

To investigate responses that included inaccurate information (score 2) or serious factual errors (score 1), we analyzed cases with an average score of <3. Among all questions, ChatGPT, Gemini, and Claude each had two items, whereas “Perplexity” had nine items with an average score below three, based on evaluations from four assessors (Table 3).

### 3.4. Comparison of Scores by Disease Across Generative AI Models

To evaluate whether the accuracy and expertise of the four AI tools differed depending on the available information level, we analyzed their performance across four diseases, ranging from extremely rare diseases with limited information to relatively common rare diseases with comparatively more information. The mean scores for the four diseases analyzed in this study showed no statistically significant differences (*p* = 0.105; Table 4).

## 4. Discussion

This study aimed to assess the accuracy of information provided by generative AI models in response to rare disease-related queries posed by patients and their families. The results showed that the four evaluated generative AI models generally provided accurate information based on reliable sources, occasionally delivering more detailed and specific responses than the intended queries. In this study, the information provided by generative AI across the different rare diseases was not significantly different. This contradicts the expectation that the scarcity of information on rare diseases leads to variations in response quality. Rare disease information is notably difficult to access using conventional sources. However, generative AI offers the advantages of speed and convenience.

However, limitations were also observed, including the presentation of incorrect information as factual, the use of complex terminology, and the inclusion of potentially confusing content. These inaccuracies vary considerably across the AI models. Among all the evaluated questions, items with an average score below three from the four evaluators included two cases each for ChatGPT, Gemini, and Claude, and nine cases for Perplexity. Examples of items rated below 2 were as follows: Claude incorrectly stated that a carrier with a 46,XX,i(21)(q10) chromosomal abnormality has a 1% chance of having a child with Down syndrome in subsequent pregnancies, despite the actual probability being close to 100% (1.00 point, Down syndrome Diagnosis-4). Perplexity erroneously suggested that prenatal treatment is available for fetuses with spinal muscular atrophy in South Korea, which is not currently possible (1.75 points, SMA Treatment-4). Additionally, Perplexity cited low-credibility sources (such as personal blogs) to erroneously describe ROHHAD syndrome as a genetic disorder (1.00 point each for ROHHAD syndrome Counseling-2 and Counseling-3). In the analysis of error types across the 15 cases from 14 questions (shown in Table 3), we identified that six cases involved the omission of critical information, while nine cases pertained to the provision of inaccurate or incorrect information, including errors arising from unreliable sources, misrepresentation of information, and the use of outdated information.

Similar issues have been reported previously. Generative AI systems, including ChatGPT, have provided incorrect information regarding genetic mechanisms, clinical diagnoses, and recurrence risk predictions, presenting such errors as factual [10]. ChatGPT demonstrates inconsistent response patterns to identical genetics-related questions, occasionally converting incorrect information into correct answers, or vice versa [10]. The model exhibited low accuracy in recurrence risk calculations, such as an incorrect estimation of the probability of having a healthy child in cases of autosomal recessive inheritance [11]. ChatGPT has also displayed “artificial hallucination” by fabricating sources or generating non-existent references, as seen in a study where two-thirds of its 59 cited references were fabricated [12].

Despite the growing interest in applying generative AI in genetic counseling, limitations in providing accurate information remain. Some genetic counselors in North America have already incorporated ChatGPT into their workflow, primarily for writing tasks. However, they remain cautious because of the risk of misinformation [13].

In this study, ChatGPT, Gemini, and Claude demonstrated higher response accuracy than Perplexity for rare-disease-related queries. Initially, Perplexity was anticipated to exhibit fewer artificial hallucinations and provide more accurate information because of its reliance on cited sources. However, contrary to these expectations, Perplexity received lower accuracy scores due to factors such as providing incorrect factual information, omitting key details, or citing low-credibility sources. This reliance on cited sources, while generally a strength, proved to be a liability when these sources lacked reliability.

Additionally, in the “Counseling” subcategory, which included questions on recurrence risks, genetic testing for children, disease-related communities and support systems, and family counseling, Perplexity performed worse than the other generative AI models. This trend of lower accuracy due to Perplexity has been noted in other studies. For instance, Perplexity underperformed ChatGPT in answering gastroenterology-related questions [14] and exhibited the lowest accuracy in complex medical decision-making scenarios [15]. However, contrasting findings exist, with some studies evaluating Perplexity as the most reliable of the five generative AI models (ChatGPT, BARD, Gemini, Copilot, and Perplexity) for citing clear and credible sources [16]. The strength of Perplexity lies in leveraging AI-powered search engines to provide real-time source-based answers [17].

Although some studies have highlighted the limitations of generative AI in providing accurate information, others have emphasized its ability to deliver reliable responses. For instance, ChatGPT has demonstrated a response accuracy comparable to that of humans (70%) and provides rapid answers to various genetic inquiries [10]. It also has high accuracy and reliability in responses related to specific conditions, such as retinopathy of prematurity [18], and provides accurate answers even in areas with prevalent misinformation, such as cancer treatment. For example, ChatGPT successfully recommends appropriate first-line therapies for specific solid tumor subtypes based on the NCCN guidelines [19]. Applications of chatbots such as Gia in the U.S.A. and Rosa in Norway have further demonstrated the utility of generative AI in collecting family histories and delivering general information to patients with hereditary breast and ovarian cancers. These tools allow healthcare providers to save time and improve consultation quality [20]. Additionally, the accessibility of chatbots, which is unconstrained by language, location, and time, leads to high user satisfaction [21]. However, limitations such as variability in ease of use depending on user demographics (such as age, income, and literacy level) have been noted [22]. Some studies have found that the lexical accuracy and readability of the information provided by generative AI may be lower than those provided by web-based sources [23], suggesting that it might present the same information in a more complex or less accessible format.

The conflicting results of the studies on the accuracy of generative AI may stem from several limitations.

First, differences in the timing of the studies may reflect variations in model performance. For example, Perplexity allows users to choose from modes tailored to web, academic, mathematical, and writing tasks. Studies using modes other than the “web mode” employed in this research may have drawn from different sources. Second, this study limited AI use to patients and their families without employing optimized prompts. The use of specialized prompts may lead to better outcomes. Finally, this study is among the first to evaluate generative AI for genetic counseling among Koreans. Unlike English, which dominates natural language processing (52.1% of digital content), Koreans account for only 0.8% [24]. Such linguistic imbalances can reflect cultural and semantic nuances inadequately, potentially leading to biased or inaccurate information [25]. Nevertheless, the Korean language queries in this study were generally handled well by the generative AI models.

**Figure 2:**
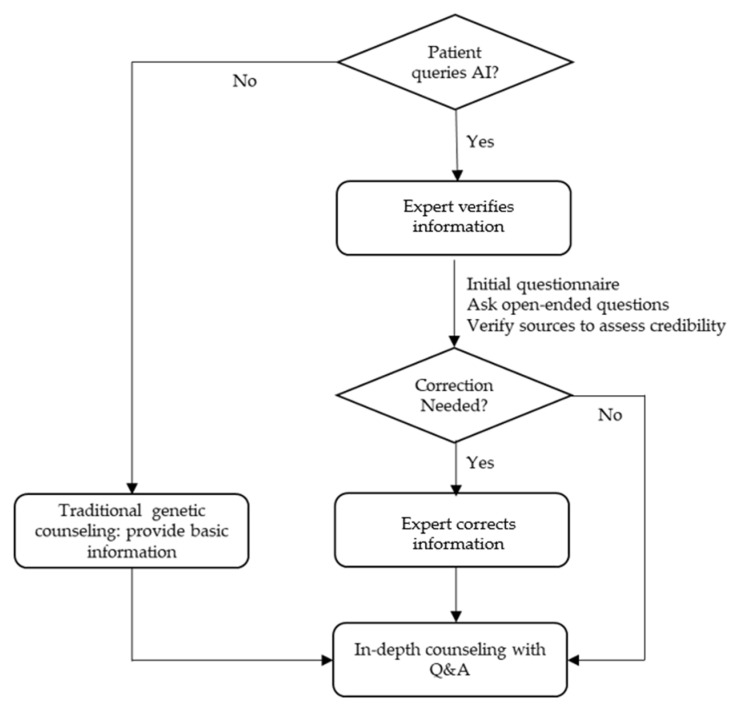
A proposed workflow integrating generative AI into genetic counseling.

At present, the safest and most effective approach is for physicians or genetic counselors, who can critically evaluate and integrate its output, to use generative AI as a resource for gathering information in the provision of genetic counseling. However, when patients or families use generative AI, the expert’s role is essential to explain the limitations of generative AI to patients and families who plan to use it and to offer guidance on safe usage practices. For example, experts should emphasize the need to compare AI-derived information with that obtained through other sources and verify the credibility of cited references. Experts should review and, if necessary, revise potentially misleading or negative information generated by AI (Figure 2).

When integrating AI into the field of genetic counseling, it is crucial to consider measures that ensure accurate information delivery. For example, providing the AI with up-to-date, high-quality data can enable more evidence-based responses, while establishing a protocol requiring expert verification for higher-risk queries may reduce misinformation. Additionally, implementing content filters to exclude potentially distressing or provocative language could help safeguard patients from unnecessary discomfort.

## 5. Conclusions

In conclusion, generative AI may serve as a supplementary tool for patients and their families seeking information, particularly when genetic counseling resources are insufficient. However, professional genetic counseling is necessary to verify the accuracy and reliability of AI-generated information.

The strengths of generative AI include its conversational capabilities and potential to improve communication and diagnostic abilities through training. With continued advancements in generative AI technology, patients with rare diseases and their families will gain easier access to more accurate and reliable information, ultimately contributing to a supplemental role in genetic counseling.

## Figures and Tables

**Table 1:** Comparison of evaluation scores by generative AI.

Generative AI	Sample Size	Mean Rank	Mean ± SD	χ^2^	df	*p* Value
ChatGPT o1-Preview	408	958.02	4.24 ± 0.73	294.061	3	<0.001
Gemini Advanced	408	911.07	4.15 ± 0.74			
Claude 3.5 sonnet	408	904.91	4.13 ± 0.82			
Perplexity Sonar Huge model (Web mode)	408	492.00	3.35 ± 0.80			

χ^2^ = Chi-square; df = Degrees of Freedom.

**Table 2:** Multiple comparison Mann–Whitney U test with Bonferroni adjustment of evaluation scores by generative AI.

Samlpe1–Sample2	Test Statistic	Standard Error	Standardized Test Statistic	*p* Value	Adjusted *p* Value *
Perplexity–Claude	412.912	31.087	13.282	<0.001	<0.001
Perplexity–Gemini	419.077	31.087	13.481	<0.001	<0.001
Perplexity–ChatGPT	466.026	31.087	14.991	<0.001	<0.001
Claude–Gemini	6.165	31.087	0.198	0.843	1.000
Claude–ChatGPT	53.114	31.087	1.709	0.088	0.525
Gemini–ChatGPT	46.949	31.087	1.510	0.131	0.786

* Bonferroni adjustment.

**Table 3:** List of responses with an average score below 3 across generative AI models.

Subcategories	Questions Utilized for Genetic Counseling with Generative AI	ChatGPT	Gemini	Claude	Perplexity
		Mean ± SD			
**Huntington’s Disease**					
Diagnosis-1	What tests and procedures are required to confirm Huntington’s disease? Do I need genetic testing?	4.50 ± 0.58	**2.50 ± 1.00**	4.25 ± 0.96	4.00 ± 0.82
Treatment-2	What progress has been made in new treatments or research for Huntington’s disease?	4.75 ± 0.50	**2.50 ± 1.00**	4.25 ± 0.50	3.75 ± 0.96
Prognosis-3	What factors can affect Huntington’s disease progression and outcome?	4.75 ± 0.50	4.50 ± 0.58	4.25 ± 0.96	**2.75 ± 0.50**
**Spinal Muscular Atrophy**					
Treatment-4	If a fetus is diagnosed with SMA, can targeted treatment begin before birth?	4.50 ± 0.58	4.50 ± 0.58	4.50 ± 0.58	**1.75 ± 0.50**
**Down Syndrome**					
General-2	Is Down syndrome classified as a rare disease?	**2.75 ± 0.96**	3.25 ± 0.50	3.75 ± 0.96	3.00 ± 0.00
General-4	What are the main genetic mechanisms of Down syndrome?	3.50 ± 0.58	4.00 ± 0.00	4.75 ± 0.50	**2.50 ± 0.58**
Diagnosis-2	Why is chromosomal testing performed when diagnosing Down syndrome?	4.00 ± 0.00	3.75 ± 0.96	4.00 ± 0.82	**2.00 ± 0.00**
Diagnosis-4	My first child’s chromosomal test shows “46,XX,i(21)(q10)”. What does this mean, and what is the likelihood of having another baby with Down syndrome in my next pregnancy?	4.00 ± 0.82	3.50 ± 0.58	**1.00 ± 0.00**	3.50 ± 1.73
Counseling-2	If my first child has Down syndrome, what are the chances my second child will also have the disease?	4.00 ± 0.82	4.25 ± 0.96	3.75 ± 0.50	**2.00 ± 0.00**
**ROHHAD syndrome**					
General-2	Is ROHHAD syndrome a rare disease?	**2.50 ± 0.58**	3.75 ± 0.50	**2.50 ± 0.58**	3.75 ± 0.96
Treatment-3	What medications or situations should patients with ROHHAD syndrome avoid?	4.50 ± 0.58	4.00 ± 1.15	3.75 ± 0.96	**2.75 ± 0.50**
Counseling-2	If my first child has ROHHAD syndrome, what are the chances my second child will also have the disease?	4.50 ± 0.58	4.25 ± 0.96	4.75 ± 0.50	**1.00 ± 0.00**
Counseling-3	Can ROHHAD syndrome patients have children, and if so, what are the risks of their children inheriting the condition?	4.25 ± 0.50	4.00 ± 0.82	3.50 ± 0.58	**1.00 ± 0.00**
Counseling-6	What institutional support is available in Korea for patients with ROHHAD syndrome or their families?	4.50 ± 0.58	4.00 ± 0.82	3.50 ± 0.58	**2.50 ± 0.58**

**Table 4:** Comparison of evaluation scores by disease.

Disease	Sample Size	Mean Rank	Mean ± SD	χ^2^	df	*p* Value
Huntington’s Disease	464	831.05	4.01 ± 0.78	6.137	3	0.105
Spinal Muscular Atrophy	416	849.96	4.04 ± 0.80			
Down Syndrome	400	798.56	3.92 ± 0.90			
ROHHAD syndrome	352	778.17	3.87 ± 0.93			

χ^2^ = Chi-square; df = Degrees of Freedom.

## Data Availability

Raw data can be obtained upon request from the corresponding author.

## Supplementary Materials

The following supporting information can be downloaded at: https://www.mdpi.com/article/10.3390/diagnostics15060672/s1, Table S1: Classification and features of Generative AI models; Table S2: Classification and features of genetic diseases; Table S3: Evaluation criteria for genetic counseling using generative AI; Table S4: Responses provided by generative AI models to each question evaluated on a Likert scale.

## Abbreviations

The following abbreviations are used in this manuscript:

AI	Artificial intelligence
df	Degrees of Freedom
IBM	International Business Machines
IRB	Institutional Review Board
KDCA	Korea Disease Control and Prevention Agency
NCCN	National Comprehensive Cancer Network
ROHHAD	Rapid-Onset Obesity with Hypothalamic Dysfunction, Hypoventilation, and Autonomic Dysregulation
SD	Standard Deviation
SMA	Spinal Muscular Atrophy
SPSS	Statistical Package for the Social Sciences
