# Supplementary material for: Evaluating the Use of Generative Artificial Intelligence to Support Genetic Counseling for Rare Diseases
Source: Diagnostics (Basel). 2025 Mar 10;15(6):672. doi: 10.3390/diagnostics15060672 (PMC11941130; doi:10.3390/diagnostics15060672)
Supplement: Supplementary file 1 [file diagnostics-15-00672-s001.zip › diagnostics-3505905-supplementary.pdf]

Table S1. Classification and features of Generative AI Models

AI type	Model used	Features
ChatGPT (Open AI□)	ChatGPT o1- Preview	- Ranked 1st in monthly visitors (March & August 2024)<br>- Most representative Large Language Model (LLM)
Gemini (Google□)	Gemini Advanced	- Ranked 2nd in monthly visitors (March 2024)<br>- Large Multi-Modal (LMM) model capable of processing various types of data
Claude (Anthropic□)	Claude 3.5 Sonnet	- Ranked 4th in monthly visitors (August 2024)<br>- The only generative AI based on ethical values
Perplexity (Perplexity AI□)	Perplexity Sonar Huge (Web mode)	- Ranked 3rd in monthly visitors (August 2024)<br>- Accurate information retrieval based on cited sources

Table S2. Classification and features of genetic diseases.

	Down Syndrome	Spinal Muscular Atrophy	Huntington's Disease	ROHHAD Syndrome
National Disease Classification	Rare disease	Rare disease	Rare disease	Extremely rare disease
National Registered Cases (2020–2021)	742	137	98	2
Prevalence	1/700–1,000	1–2/100,000	1–5/100,000	<1/1,000, 000
Age of onset	At birth	From birth to adulthood (varies by SMA type)	Adulthood (30–50 years)	Early childhood (1–10 years)
Median age at death	Around 50 years	6 months– early adulthood (varies by SMA type)	50–70 years	Around 10 years
Cause	Abnormality in chromosome 21	*SMN1* gene exon deletion (autosomal recessive)	*HTT* gene trinucleotide repeat expansion (autosomal dominant)	Unknown
Genetic Diagnosis	Karyotyping, FISH, QF-PCR etc.	MLPA, qPCR	Expanded repeat analysis etc.	–
Treatment	Symptom-based management	Symptom-based management, medications (Zolgensma, Risdiplam, etc.)	Symptom-based management	Symptom-based management

ROHHAD = Rapid-Onset Obesity with Hypothalamic Dysfunction, Hypoventilation, and Autonomic Dysregulation; FISH = Fluorescence in Situ Hybridization; QF-PCR = Quantitative fluorescence-polymerase chain reaction; qPCR = Quantitative polymerase chain reaction;

Table S3. Evaluation criteria for genetic counseling using generative AI

Score	Professionalism	Information Accuracy	Impact on Patients and Families
5: Excellent	Highly professional; includes additional information and thorough explanation.	Perfectly accurate; no errors or inconsistencies.	Provides patients and families with useful, stress-free, and reliable psychological support.
4: Good	Professional; adequately thorough.	Highly accurate; minor factual errors or omissions.	Minimal stress or confusion for patients and families; almost no negative impact.
3: Fair	Basic professionalism; adequate content but room for improvement.	Moderately accurate; some factual gaps or inaccuracies.	Minor confusion or stress for patients and families; tolerable impact.
2: Poor	Lacking professionalism; includes inappropriate or poorly explained information.	Inaccurate information present.	Noticeable confusion or stress for patients and families; harmful impact.
1: Very Poor	Severely unprofessional; critical omissions or misleading information.	Serious factual errors present.	High risk of stress or harm to patients and families; critical negative impact.

Table S4. Responses provided by Generative AI models to each question evaluated on a Likert scale

Sub-categories	Questions Utilized for Genetic Counseling with Generative AI [a]	ChatGPT	Gemini	Claude	Perplexity
		Mean ± SD			
	Huntington's Disease (29 questions)				
General-1	헌팅턴병이 어떤 질환인지 설명해 주세요. (Please explain what Huntington's disease is.)	3.75±0.96	4.25±0.50	4.50±0.58	4.00±0.82
General-2	헌팅턴병은 희귀질환인가요? (Is Huntington's disease a rare disease?)	3.50±1.00	3.50±1.00	4.50±0.58	4.50±0.58
General-3	헌팅턴병이 발생하는 원인은 무엇인가요? (What causes Huntington's disease?)	3.75±0.50	4.00±0.00	4.75±0.50	3.75±0.50
General-4	헌팅턴병의 유전 양상은 어떤가요? (What is the inheritance pattern of Huntington's disease?)	4.00±0.82	3.50±0.58	4.50±1.00	4.00±0.00
General-5	헌팅턴병에서 나타나는 주요 장애는 무엇입니까? (What are the main impairments associated with Huntington's disease?)	4.50±0.58	4.50±0.58	3.50±0.58	3.00±0.00
General-6	헌팅턴병을 가질 위험을 높이는 유전적인 이유나 다른 위험 요인이 있나요? (Are there genetic reasons or other risk factors that increase the likelihood of Huntington's disease?)	4.00±0.00	3.75±0.50	4.25±0.50	3.50±1.00
General-7	헌팅턴병을 진단받게 되는 사람들에게서 흔히 나타나는 초기 증상은 무엇입니까? (What are the early symptoms commonly seen in people diagnosed with Huntington's disease?)	4.25±0.50	4.50±0.58	4.25±0.96	3.50±0.58
Diagnosis-1	헌팅턴병을 확진하려면 어떤 검사와 절차가 필요한가요? 유전자 검사를 해야 하나요? (What tests and procedures are required to confirm Huntington's disease? Do I need genetic testing?)	4.50±0.58	2.50±1.00	4.25±0.96	4.00±0.82
Diagnosis-2	헌팅턴병을 진단할 때 유전자검사를 하는 이유는 무엇인가요? (Why is genetic testing performed when diagnosing Huntington's disease?)	4.50±1.00	4.50±0.58	4.75±0.50	4.25±0.96

Diagnosis-3	헌팅턴병 유전자검사 결과에서 나는 헌팅턴병이 나타나지 않지만 자녀는 헌팅턴병이 발병할 수 있다고 들었습니다. 이런 상황이 가능한가요? (I was told that my child could still develop Huntington's disease even if it doesn't appear in my genetic test. Is this true?)	4.50±0.58	4.00±0.00	4.50±0.58	3.25±0.50
Diagnosis-4	유전자검사 결과지에 적힌 "Number of CAG Trinucleotide Repeats : 41"은 무슨 뜻인가요? (What does "Number of CAG Trinucleotide Repeats: 41" on my genetic test report mean?)	4.25±0.96	3.50±0.58	4.25±0.96	3.25±0.50
Diagnosis-5	헌팅턴병의 중증도를 어떻게 판단하나요? (How is the severity of Huntington's disease determined?)	4.50±0.58	4.50±0.58	4.25±0.96	3.00±0.00
Treatment-1	헌팅턴병 치료와 관리는 어떻게 하나요? (How is Huntington's disease treated and managed?)	4.50±0.58	4.25±0.50	3.50±0.58	3.00±0.00
Treatment-2	헌팅턴병에 대한 새로운 치료법이나 연구는 어떻게 진행되고 있나요? (What progress has been made in new treatments or research for Huntington's disease?)	4.75±0.50	2.50±1.00	4.25±0.50	3.75±0.96
Treatment-3	헌팅턴병을 근본적으로 치료할 수 있는 방법이 있나요? (Is there a way to fundamentally cure Huntington's disease?)	4.25±0.50	4.50±0.58	4.00±0.82	4.00±0.82
Treatment-4	헌팅턴병 환자에서 피해야 할 약물이나 상황은 무엇인가요? (What medications or situations should be avoided in patients with Huntington's disease?)	4.75±0.50	4.25±0.50	4.00±0.82	3.00±0.00
Treatment-5	헌팅턴병에 대해 FDA 승인을 받은 약물이 있나요? (Are there any FDA-approved drugs for Huntington's disease?)	4.25±0.50	4.50±0.58	4.25±0.50	4.25±0.96
Prognosis-1	헌팅턴병은 환자에게 장기적으로 호전되거나 악화될 수 있나요? (Can Huntington's disease improve or worsen over the long term for patients?)	4.50±0.58	3.50±0.58	4.00±0.82	3.50±0.58
Prognosis-2	헌팅턴병이 발병한 후 일반적인 기대수명과 사망 중앙연령은 몇 살인가요? (What is the general life expectancy and median age of death after Huntington's disease onset?)	3.75±0.50	3.75±0.50	4.25±0.96	3.50±0.58
Prognosis-3	헌팅턴병에서 질병 진행 속도와 예후에 영향을 줄 수 있는 요인은 무엇인가요? (What factors can affect Huntington’s disease progression and outcome?)	4.75±0.50	4.50±0.58	4.25±0.96	2.75±0.50
Counseling-1	나는 헌팅턴병으로 진단받았습니다. 내 자녀가 같은 질환을 가지고 있을 확률과 형제자매들이 같은 질환을 가질 확률은 각각 얼마나 되나요? (I have been diagnosed with Huntington's disease. What are the chances my child or siblings have the same disease?)	4.25±0.50	4.00±0.00	4.25±0.96	3.50±0.58

Counseling-2	나는 헌팅턴병으로 진단받았습니다. 자녀는 헌팅턴병 유전자 검사에서 음성이 나왔어도 헌팅턴병이 발생할 수 있나요? (I have been diagnosed with Huntington's disease. Can my child develop the disease even if the genetic test came back negative?)	5.00±0.00	3.50±0.58	4.75±0.50	3.25±0.50
Counseling-3	나는 헌팅턴병으로 진단받았습니다. 다음 번 임신할 때 체외 수정을 하게 된다면 배아를 대상으로 헌팅턴병 유전자 검사를 받을 수 있나요? (I have been diagnosed with Huntington's disease. Can I test an embryo for Huntington's disease genes during IVF for my next pregnancy?)	4.25±0.50	4.50±0.58	4.00±0.82	3.25±0.50
Counseling-4	나는 헌팅턴병으로 진단받았습니다. 올해로 15 세가 된 내 아들도 유전자 검사를 할 수 있나요? (I have been diagnosed with Huntington's disease. Can my 15-year-old son get a genetic test?)	4.50±1.00	3.00±0.00	4.25±0.50	3.50±0.58
Counseling-5	헌팅턴병 환자를 돕기 위해 가족들은 무엇을 해야 하나요? (What should family members do to help patients with Huntington's disease?)	4.50±0.58	4.50±0.58	4.25±0.96	3.25±0.50
Counseling-6	헌팅턴병 환자는 살아가면서 어떤 증상이 가장 힘들고 문제가 되나요? (What are the most challenging symptoms for patients with Huntington's disease to live with?)	4.50±0.58	4.25±0.50	4.00±0.82	3.75±0.96
Counseling-7	헌팅턴병 환자나 가족을 위한 우리나라(대한민국)의 제도적 지원에는 무엇이 있나요? (What institutional support is available in Korea for patients with Huntington's disease or their families?)	4.50±0.58	3.75±0.50	4.00±0.82	3.00±0.00
Counseling-8	내가 헌팅턴병 진단받았다는 사실을 가족에게 어떻게 설명하면 좋을까요? (How should I explain my Huntington's disease diagnosis to my family?)	4.25±0.96	4.50±1.00	4.00±0.82	3.50±0.58
Counseling-9	우리나라(대한민국)에 헌팅턴병 환자나 가족들을 위한 모임이나 커뮤니티가 있나요? (Are there any groups or communities for patients with Huntington's disease or their families in Korea?)	4.50±0.58	4.25±0.50	4.00±0.82	3.50±0.58
Spinal Muscular Atrophy (26 questions)					
General-1	척수성 근위축증이 어떤 질환인지 설명해 주세요. (Please explain what spinal muscular atrophy (SMA) is.)	3.50±0.58	4.50±0.58	3.25±0.50	4.00±0.82
General-2	척수성 근위축증은 희귀질환인가요? (Is SMA a rare disease?)	3.00±0.00	4.00±0.00	4.25±0.96	3.50±0.58

General-3	척수성 근위축증이 발생하는 원인과 메커니즘은 무엇인가요? (What causes SMA, and what is its mechanism?)	4.00±0.00	4.50±0.58	4.50±1.00	4.25±0.96
General-4	척수성 근위축증 질환의 유전방식은 어떻게 되나요? (What is the inheritance pattern of SMA?)	4.75±0.50	4.25±0.96	4.25±0.96	4.00±1.15
General-5	척수성 근위축증에 여러 유형이 있다고 들었는데, 구체적으로 설명해 주세요 (I've heard there are several types of SMA. Can you explain them in detail?)	4.25±0.50	4.50±0.58	4.50±1.00	4.25±0.96
General-6	척수성 근위축증을 진단받은 경우 연령대별로 특히 조심해야 하는 점은 무엇인가요? (What should individuals diagnosed with SMA be particularly cautious of at different ages?)	4.75±0.50	4.75±0.50	4.50±0.58	3.25±0.50
Diagnosis-1	척수성 근위축증을 정확하게 진단받으려면 어떤 검사와 절차가 필요한가요? (What tests and procedures are required for an accurate diagnosis of SMA?)	4.25±0.96	3.75±0.96	4.75±0.50	3.25±0.50
Diagnosis-2	척수성 근위축증을 진단할 때 유전자검사를 하는 이유는 무엇인가요? (Why is genetic testing performed for SMA diagnosis?)	4.75±0.50	4.25±0.96	4.50±1.00	3.25±0.50
Diagnosis-3	SMA 유전자 결손 또는 중복 검사 시 거짓 음성 확률이 있다는 의미는 무엇인가요? (What does it mean that there is a false-negative probability for SMA gene deletion or duplication tests?)	4.25±0.50	4.25±0.50	4.00±0.82	4.00±0.82
Diagnosis-4	SMA 유전자 검사에서 양성으로 나왔어도 척수성 근위축증이 아닐 수 있나요? (Is it possible to have a positive SMA genetic test result but not have the disease?)	4.50±0.58	4.25±0.96	4.25±0.96	4.00±0.82
Diagnosis-5	유전자 검사결과에 따라 질환의 중증도에 차이가 있을 수 있나요? (Can the severity of SMA vary based on genetic test results?)	4.25±0.50	4.00±0.00	4.25±0.96	3.50±0.58
Diagnosis-6	"SMN1 homozygous deletion" 라고 유전자결과가 나왔는데, 이것은 어떻게 해석하면 되나요? (What does the genetic result "SMN1 homozygous deletion" mean?)	4.50±0.58	4.25±0.96	4.50±1.00	4.00±0.82
Treatment-1	척수성 근위축증 치료와 관리는 어떻게 하나요?(How is SMA treated and managed?)	4.50±0.58	4.75±0.50	4.00±0.82	3.25±0.50
Treatment-2	척수성 근위축증에 대한 새로운 치료법과 연구는 어떻게 진행되고 있나요? (What progress has been made in new treatments or research for SMA?)	4.25±0.50	4.00±0.00	4.00±0.82	3.00±0.00
Treatment-3	척수성 근위축증 치료는 어느 시기에 시작하는 것이 좋은가요? (When is the best time to start treatment for SMA?)	4.25±0.50	3.75±0.50	4.50±0.58	3.00±0.00

Treatment-4	만약 척수성 근위축증을 가진 태아를 임신한다면 출산 전에 미리 표적 치료를 시작할 수 있나요? (If a fetus is diagnosed with SMA, can targeted treatment begin before birth?)	4.50±0.58	4.50±0.58	4.50±0.58	1.75±0.50
Prognosis-1	척수성 근위축증이 발병한 후 일반적인 기대수명과 환자의 사망 중앙연령은 몇 살인가요? (What is the general life expectancy and median age of death after SMA onset?)	4.25±0.96	4.50±0.58	4.50±0.58	3.75±0.96
Prognosis-2	척수성 근위축증에서 질병 진행 속도에 영향을 줄 수 있는 요인은 무엇인가요? (What factors can influence SMA progression?)	4.50±0.58	4.00±0.82	4.25±0.50	3.00±0.00
Counseling-1	자녀가 척수성 근위축증으로 진단받았습니다. 부모 중 누구에게 척수성 근위축증을 물려받은 건가요? (My child has been diagnosed with SMA. Which parent passed on the condition?)	4.25±0.50	3.50±0.58	4.25±0.50	3.25±0.50
Counseling-2	첫 자녀가 척수성 근위축증이었다면 둘째 아이가 같은 질환을 가지고 태어날 확률은 얼마나 되나요? (If my first child has SMA, what are the chances my second child will also have the disease?)	4.25±0.50	3.25±0.50	4.00±0.82	3.50±0.58
Counseling-3	자녀가 척수성 근위축증으로 진단받았습니다. 다음 번 임신할 때 체외수정을 하게 된다면 배아를 대상으로 척수성 근위축증 유전자 검사를 받을 수 있나요? (My child has been diagnosed with SMA. Can I test embryos for SMA genes during IVF for my next pregnancy?)	4.50±0.58	4.50±0.58	4.00±0.82	3.25±0.50
Counseling-4	자녀가 척수성 근위축증으로 진단받았습니다. 올해로 3 세가 된 조카도 유전자 검사를 할 수 있나요? (My child has been diagnosed with SMA. Can my 3-year-old nephew get a genetic test?)	4.50±0.58	3.75±0.50	4.25±0.50	3.00±0.00
Counseling-5	결혼할 상대가 척수성 근위축증 원인 유전자의 보인자인지 결혼 전에 미리 검사할 수 있나요? (Can I test if my fiancé is a carrier of the gene causing SMA before marriage?)	4.50±0.58	4.50±0.58	4.50±0.58	3.00±0.00
Counseling-6	우리나라(대한민국)에 척수성 근위축증 환자나 가족들을 위한 모임이나 커뮤니티가 있나요? (Are there any groups or communities for patients with SMA or their families in Korea?)	4.25±0.50	3.75±1.26	4.25±0.96	3.25±0.50
Counseling-7	척수성 근위축증 환자나 가족을 위한 우리나라(대한민국)의 제도적 지원에는 무엇이 있나요? (What institutional support is available in Korea for patients with SMA or their families?)	4.50±0.58	4.00±0.00	4.00±0.82	3.00±0.00

Counseling-8	자녀가 척수성 근위축증을 진단받았다는 사실을 가족에게 어떻게 설명하면 좋을까요?<br>(How should I explain my child's SMA diagnosis to my family?)	4.75±0.50	4.75±0.50	4.25±0.96	3.25±0.50
	Down Syndrome (25 questions)				
General-1	다운증후군이 어떤 질환인지 설명해 주세요. (Please explain what Down syndrome is.)	3.00±0.00	4.00±0.00	4.00±0.00	3.75±0.50
General-2	다운증후군은 희귀질환인가요? (Is Down syndrome classified as a rare disease?)	2.75±0.96	3.25±0.50	3.75±0.96	3.00±0.00
General-3	다운증후군이 발생하는 원인은 무엇인가요? (What causes Down syndrome?)	3.00±0.00	4.50±0.58	4.25±0.50	3.75±0.50
General-4	다운증후군 질환의 유전방식은 무엇인가요? (What are the main genetic mechanisms of Down syndrome?)	3.50±0.58	4.00±0.00	4.75±0.50	2.50±0.58
General-5	다운증후군 아이를 출산할 위험을 높이는 요인은 무엇인가요?<br>(What factors increase the risk of giving birth to a child with Down syndrome?)	3.50±0.58	4.50±0.58	4.00±0.82	3.00±0.00
Diagnosis-1	산전 검사에서 '다운증후군 고위험군' 결과가 확인되었습니다. 이 결과는 어떤 의미인가요?<br>(What does it mean if a prenatal test shows a "high risk of Down syndrome"?)	4.50±0.58	4.75±0.50	4.25±0.96	3.75±0.96
Diagnosis-2	다운증후군을 진단할 때 염색체검사를 하는 이유는 무엇인가요?<br>(Why is chromosomal testing performed when diagnosing Down syndrome?)	4.00±0.00	3.75±0.96	4.00±0.82	2.00±0.00
Diagnosis-3	모체 혈액을 이용한 NIPT 검사 결과가 음성으로 나왔어도 다운증후군 아이를 출산할 수 있나요?<br>(Can a Down syndrome baby still be born even if the NIPT results are negative?)	4.00±0.82	4.50±1.00	4.50±0.58	3.00±0.00
Diagnosis-4	첫번째 아기 염색체 검사 결과지에 "46,XX,i(21)(q10)" 라고 적혀 있습니다. 이것은 무슨 뜻인가요? 또한 다음 임신 시 다운증후군 아기를 출산할 확률은 얼마인가요?<br>(My first child's chromosomal test shows "46,XX,i(21)(q10)". What does this mean, and what is the likelihood of having another baby with Down syndrome in my next pregnancy?)	4.00±0.82	3.50±0.58	1.00±0.00	3.50±1.73
Treatment-1	다운증후군 치료와 관리는 어떻게 하나요? (How is Down syndrome treated and managed?)	5.00±0.00	4.50±0.58	4.25±0.96	3.50±0.58
Treatment-2	다운증후군에 대한 새로운 치료법이나 연구는 어떻게 진행되고 있나요?<br>(What progress has been made in new treatments or research for Down syndrome?)	4.25±0.96	4.75±0.50	4.75±0.50	3.50±0.58

Treatment-3	다운증후군을 근본적으로 치료할 수 있는 방법이 있나요? (Is there a way to fundamentally cure Down syndrome?)	3.50±0.58	5.00±0.00	4.75±0.50	3.50±0.58
Prognosis-1	다운증후군이 발병한 후 일반적인 기대수명과 사망 중앙연령은 몇 살인가요? (What is the general life expectancy and median age of death after Down syndrome onset?)	3.50±0.58	4.75±0.50	4.25±0.50	3.00±0.00
Prognosis-2	다운증후군에서 질병 진행 속도와 예후에 영향을 줄 수 있는 요인은 무엇인가요? (What factors can influence Down syndrome progression and prognosis?)	4.25±0.50	4.50±1.00	4.25±0.96	3.00±0.82
Prognosis-3	다운증후군 환자에게 발생할 확률이 높은 합병증은 무엇이며, 어떻게 관리하여야 하나요? (What are the common complications in patients with Down syndrome , and how can they be managed?)	4.50±1.00	4.25±0.50	4.00±0.82	3.25±0.50
Counseling-1	자녀가 다운증후군으로 진단받았습니다. 부모 중 누구에게 다운증후군을 물려받은 건가요? (My child has been diagnosed with Down syndrome. Which parent passed on the condition?)	4.25±0.50	4.25±0.50	4.25±0.96	3.50±0.58
Counseling-2	첫 자녀가 다운증후군이었다면 둘째 아이가 같은 질환을 가지고 태어날 확률은 얼마나 되나요? (If my first child has Down syndrome, what are the chances my second child will also have the disease?)	4.00±0.82	4.25±0.96	3.75±0.50	2.00±0.00
Counseling-3	다운증후군 환자가 자녀를 낳을 수 있나요? 그렇다면 같은 질환을 가진 아이가 태어날 위험율은 얼마나 높은가요? (Can patients with Down syndrome have children, and if so, what are the risks of their children inheriting the condition?)	4.50±0.58	4.50±0.58	4.25±0.96	3.25±0.50
Counseling-4	엄마의 나이가 35 세 미만으로 고령이 아님에도 다운증후군을 가진 아이가 태어나는 이유는 무엇인가요? (Why is it possible for a child with Down syndrome to be born even if the mother is not of advanced age under 35?)	4.25±0.50	4.25±0.50	3.75±0.96	3.25±0.50
Counseling-5	다운증후군 환자는 살아가면서 어떤 증상이 가장 힘들고 문제가 되나요? 이를 돕기 위해 가족들은 무엇을 할 수 있나요? (What are the most challenging symptoms for patients with Down syndrome to live with, and how can family members help?)	4.50±0.58	4.75±0.50	4.25±0.96	3.75±0.50
Counseling-6	우리나라(대한민국)에 다운증후군 환자나 가족들을 위한 모임이나 커뮤니티가 있나요? (Are there any groups or communities for patients with Down syndrome or their families in	4.25±0.96	4.00±0.82	4.25±0.96	3.00±0.00

	Korea?)				
Counseling-7	다운증후군 환자나 가족을 위한 우리나라(대한민국)의 제도적 지원에는 무엇이 있나요? (What institutional support is available in Korea for patients with Down syndrome or their families?)	4.25±0.50	4.75±0.50	4.25±0.96	3.00±0.00
Counseling-8	자녀가 다운증후군을 진단받았다는 사실을 가족에게 어떻게 설명하면 좋을까요? (How should I explain my child's Down syndrome diagnosis to my family?)	4.75±0.50	5.00±0.00	4.25±0.50	3.50±0.58
Counseling-9	다운증후군 아이는 학교를 다니거나 연령에 따른 적절한 교육을 받을 수 있나요? (Can children with Down syndrome go to school and receive age-appropriate education?)	4.75±0.50	4.25±0.96	4.50±0.58	3.00±0.00
Counseling-10	다운증후군 자녀가 독립적으로 생활할 수 있도록 능력개발과 직업훈련을 받으려면 어떻게 해야 하나요? (How can I help my child with Down syndrome develop independence and receive vocational training?)	4.75±0.50	4.50±0.58	4.00±0.82	3.25±0.50
	ROHHAD syndrome (22 questions)				
General-1	로하드증후군이 어떤 질환인지 설명해 주세요. (Please explain what ROHHAD syndrome is.)	4.25±0.50	4.50±0.58	3.75±0.50	4.25±0.96
General-2	로하드증후군은 희귀질환인가요? (Is ROHHAD syndrome a rare disease?)	2.50±0.58	3.75±0.50	2.50±0.58	3.75±0.96
General-3	로하드증후군이 발생하는 원인은 무엇인가요? (What causes ROHHAD syndrome?)	4.00±0.82	4.50±0.58	4.50±0.58	3.50±0.58
General-4	로하드증후군을 가질 위험을 높이는 요인은 무엇인가요? (What factors increase the likelihood of developing ROHHAD syndrome?)	4.00±0.82	4.00±0.82	3.25±0.50	3.75±0.96
Diagnosis-1	로하드증후군으로 확진 받으려면 어떤 검사와 절차가 필요한가요? 유전자 검사를 해야 하나요? (What tests and procedures are required to diagnose ROHHAD syndrome? Is genetic testing necessary?)	4.25±0.96	3.75±0.50	4.25±0.96	3.25±0.50
Diagnosis-2	로하드증후군과 감별해야 하는 질환은 무엇이며, 이 질환들은 로하드증후군과 어떤 차이점이 있나요? (What diseases should be differentiated from ROHHAD syndrome, and how are they different?)	4.75±0.50	3.00±0.82	4.50±0.58	3.75±0.96

Diagnosis-3	로하드증후군을 가진 자녀의 부모가 유전자검사를 한다면 원인을 찾는 일에 도움이 되나요? (If parents of a child with ROHHAD syndrome undergo genetic testing, can it help identify the cause?)	4.25±0.96	4.25±0.96	4.25±0.50	3.50±1.00
Diagnosis-4	로하드증후군 중등도를 판단하는 요소들은 어떤 것이 있나요? (What factors are used to assess the severity of ROHHAD syndrome?)	4.25±0.50	4.50±0.58	3.75±0.50	3.75±0.50
Treatment-1	로하드증후군 치료와 관리는 어떻게 하나요? (How is ROHHAD syndrome treated and managed?)	4.50±0.58	4.50±0.58	3.75±0.96	3.50±0.58
Treatment-2	로하드증후군에 대한 새로운 치료법이나 연구는 어떻게 진행되고 있나요? (What progress has been made in new treatments or research for ROHHAD syndrome?)	3.50±1.00	4.00±0.82	3.75±0.96	3.25±0.96
Treatment-3	환자가 피해야 할 약물이나 상황은 무엇인가요? (What medications or situations should patients with ROHHAD syndrome avoid?)	4.50±0.58	4.00±1.15	3.75±0.96	2.75±0.50
Prognosis-1	로하드증후군은 환자에게 장기적으로 호전되거나 악화될 수 있나요? (Can ROHHAD syndrome improve or worsen over the long term for patients?)	4.50±0.58	4.25±0.50	4.00±0.82	3.00±0.00
Prognosis-2	로하드증후군이 발병한 후 일반적인 기대수명과 사망 중앙연령은 몇 살인가요? (What is the general life expectancy and median age of death after ROHHAD syndrome onset?)	3.75±0.96	3.75±0.96	3.75±0.96	3.25±0.50
Prognosis-3	로하드증후군에서 질병 진행 속도와 예후에 영향을 줄 수 있는 요인은 무엇인가요? (What factors can influence ROHHAD syndrome progression and prognosis?)	4.00±0.82	4.00±0.82	4.00±0.82	3.50±0.58
Counseling-1	자녀가 로하드증후군으로 진단받았습니다. 부모 중 누구에게 로하드증후군을 물려받은 건가요? (My child has been diagnosed with ROHHAD syndrome. Which parent passed on the condition?)	4.00±1.41	4.00±0.82	4.25±0.50	3.50±0.58
Counseling-2	첫 자녀가 로하드증후군이었다면 둘째 아이가 같은 질환을 가지고 태어날 확률은 얼마나 되나요? (If my first child has ROHHAD syndrome, what are the chances my second child will also have the disease?)	4.50±0.58	4.25±0.96	4.75±0.50	1.00±0.00
Counseling-3	로하드증후군 환자가 자녀를 낳을 수 있나요? 그렇다면 같은 질환을 가진 아이가 태어날 위험율은 얼마나 높은가요? (Can ROHHAD syndrome patients have children, and if so, what are the risks of their children inheriting the condition?)	4.25±0.50	4.00±0.82	3.50±0.58	1.00±0.00

Counseling-4	로하드증후군 환자는 살아가면서 어떤 증상이 가장 힘들고 문제가 되나요? 이를 돕기 위해 가족들은 무엇을 할 수 있나요? (What are the most challenging symptoms for patients with ROHHAD syndrome to live with, and how can family members help?)	4.50±0.58	4.50±0.58	4.00±1.15	4.00±0.82
Counseling-5	우리나라(대한민국)에 로하드증후군 환자나 가족들을 위한 모임이나 커뮤니티가 있나요? (Are there any groups or communities for patients with ROHHAD syndrome or their families in Korea?)	4.75±0.50	4.25±0.50	4.00±0.82	3.25±0.50
Counseling-6	로하드증후군 환자나 가족을 위한 우리나라(대한민국)의 제도적 지원에는 무엇이 있나요? (What institutional support is available in Korea for patients with ROHHAD syndrome or their families?)	4.50±0.58	4.00±0.82	3.50±0.58	2.50±0.58
Counseling-7	자녀가 로하드증후군을 진단받았다는 사실을 가족 에게 어떻게 설명하면 좋을까요? (How should I explain my child's ROHHAD syndrome diagnosis to my family?)	4.75±0.50	4.75±0.50	4.50±0.58	3.75±0.96
Counseling-8	로하드증후군 아이는 학교를 다니거나 연령에 따른 적절한 교육을 받을 수 있나요? (Can children with ROHHAD syndrome attend school or receive appropriate age-based education?)	4.25±0.96	4.25±0.96	4.00±0.82	3.25±0.96

[a] Each question was provided in Korean for generative AI.
